# Automated Quantification of Brain Lesion Volume From Post-trauma MR Diffusion-Weighted Images

**DOI:** 10.3389/fneur.2021.740603

**Published:** 2022-02-23

**Authors:** Thomas Mistral, Pauline Roca, Christophe Maggia, Alan Tucholka, Florence Forbes, Senan Doyle, Alexandre Krainik, Damien Galanaud, Emmanuelle Schmitt, Stéphane Kremer, Adrian Kastler, Irène Troprès, Emmanuel L. Barbier, Jean-François Payen, Michel Dojat

**Affiliations:** ^1^Univ. Grenoble Alpes, Inserm U1216, CHU Grenoble Alpes, Grenoble Institut Neurosciences, Grenoble, France; ^2^Pixyl, Grenoble, France; ^3^Univ. Grenoble Alpes, Inria, CNRS, Grenoble INP, LJK, Grenoble, France; ^4^Univ. Grenoble Alpes, Inserm, CHU Grenoble Alpes, CNRS, IRMaGe, Grenoble, France; ^5^APHP, Hôpital Pitié Salpétrière, Paris, France; ^6^CHU, Hôpital Central, Nancy, France; ^7^CHU, de Strasbourg, Strasbourg, France

**Keywords:** MRI, mean diffusion (MD), segmentation (image processing), traumatic brain injury, brain

## Abstract

**Objectives:**

Determining the volume of brain lesions after trauma is challenging. Manual delineation is observer-dependent and time-consuming and cannot therefore be used in routine practice. The study aimed to evaluate the feasibility of an automated atlas-based quantification procedure (AQP) based on the detection of abnormal mean diffusivity (MD) values computed from diffusion-weighted MR images.

**Methods:**

The performance of AQP was measured against manual delineation consensus by independent raters in two series of experiments based on: (i) realistic trauma phantoms (*n* = 5) where low and high MD values were assigned to healthy brain images according to the intensity, form and location of lesion observed in real TBI cases; (ii) severe TBI patients (*n* = 12 patients) who underwent MR imaging within 10 days after injury.

**Results:**

In realistic TBI phantoms, no statistical differences in Dice similarity coefficient, precision and brain lesion volumes were found between AQP, the rater consensus and the ground truth lesion delineations. Similar findings were obtained when comparing AQP and manual annotations for TBI patients. The intra-class correlation coefficient between AQP and manual delineation was 0.70 in realistic phantoms and 0.92 in TBI patients. The volume of brain lesions detected in TBI patients was 59 ml (19–84 ml) (median; 25–75th centiles).

**Conclusions:**

Our results support the feasibility of using an automated quantification procedure to determine, with similar accuracy to manual delineation, the volume of low and high MD brain lesions after trauma, and thus allow the determination of the type and volume of edematous brain lesions. This approach had comparable performance with manual delineation by a panel of experts. It will be tested in a large cohort of patients enrolled in the multicenter OxyTC trial (NCT02754063).

## Key Points

- The management of patients with severe (Glasgow coma score <9) traumatic brain injury is complex, and access to objective quantitative information regarding lesion volumes can support clinical decision-making.
- An automated delineation procedure was developed to determine the low and high MD abnormality brain lesion volumes post-trauma.
- Automated brain lesion typing and volume quantification compared favorably with manual delineation by a panel of experts.

## Introduction

Traumatic brain injury (TBI) remains a leading cause of death and disability among individuals. Only a small proportion of patients with severe TBI, as defined by an initial Glasgow Coma Scale (GCS) score of <9, will have no disabilities (1). Predicting neurological outcome after severe TBI is challenging due to the complexity of the traumatic lesion, its evolution over time, and the number of external factors that may affect the outcome. Nevertheless, determining the type and volume of brain lesion have been identified as clinically relevant criteria in estimating outcome (2).

Data are very limited concerning the use of automated methods to quantify brain injury post-trauma (3, 4). Skull deformation, intracranial blood in the brain tissue, the presence of cerebrospinal fluid (CSF) and the heterogeneity of brain tissue injury make the segmentation of traumatic brain lesions challenging. Automated approaches using non-contrast CT imaging were developed for cranial cavity segmentation (5), cistern segmentation or detection of intracranial hematomas (6). More intracranial lesions (e.g., brain swelling or intracranial hemorrhage) can be detected however by MRI, due to its higher sensitivity (2).

Diffusion-weighted imaging (DWI) is a sensitive technique for detecting subtle microstructural changes in white matter tracts, and is particularly suitable for identifying edema and necrosis (7, 8). While a reduction of mean diffusivity (MD) is indicative of cellular (cytotoxic) edema, an increase indicates a vasogenic edema (3). Both types of brain edema exist at the acute phase (<15 days after injury) of severe TBI, and are major contributors to the elevation of intracranial pressure and poor outcome after TBI (9). A good concordance was shown between DWI and clinical prognosis scores in TBI patients (4).

Our aim was to develop an automated approach to type and quantify post-traumatic edematous brain lesion volumes using MD values from DWI. The test of the feasibility was performed using both realistic digital TBI phantoms, i.e., DWI volumes from healthy subjects where realistic low and high MD values were manually introduced by a neuroradiologist, and MR images of severe TBI patients. Automated delineation results were compared against those from manual delineations performed by expert raters.

## Methods

**Figure 1:**
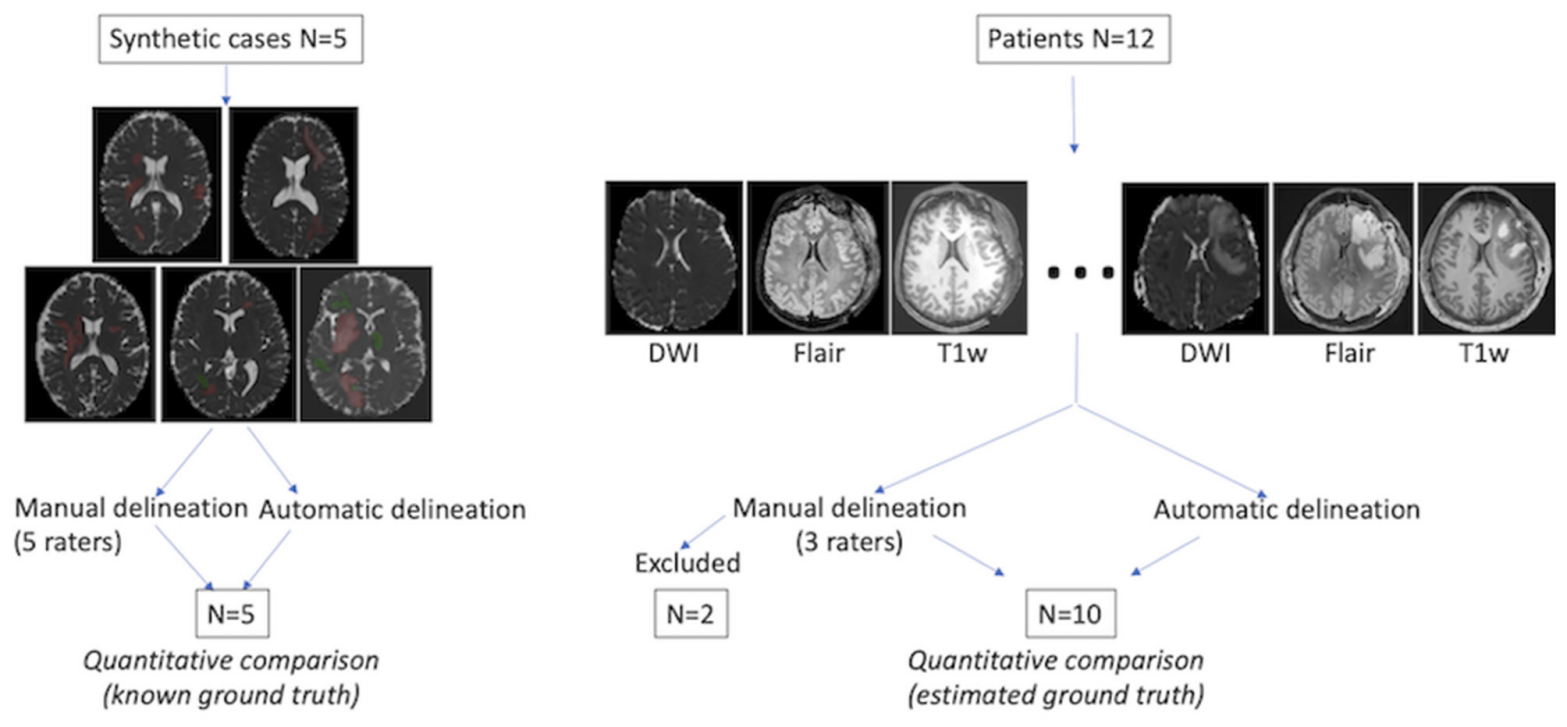
Evaluation procedure. **Left**: five realistic TBI lesion cases were constructed with low (green) and high (red) artificial MD values. The ground truth was predefined for automated and manual lesion delineation comparison. **Right**: Twelve TBI patients were included, each with three types of MR image. Manual and automated delineation results were quantitatively compared for 10 patients. The ground truth was defined as the consensus of expert annotations (“consensual inter-raters ground truth”), calculated using STAPLE (10).

Two sets of experiments were performed based on (Figure 1): (i) realistic TBI phantoms comprising artificially introduced lesions with abnormal MD values; and (ii) MR images of TBI patients included in an ongoing multicenter clinical trial (OxyTC, NCT02754063) to validate MRI acquisition. Manual delineation was performed by a panel of five expert neuroradiologists.

### Realistic TBI Phantoms

DWI was performed on five healthy volunteers (Philips Achieva 3.0T TX, Philips Healthcare, Best, Netherlands) at the IRMaGe MRI facility (Grenoble, France). Low and high MD values, simulating cellular and vasogenic brain edema, respectively, were manually inserted in these brain images by a neuroradiologist (TM) familiar with traumatic lesions. The simulated values were obtained by the application of a multiplicative coefficient to the real MD values. The coefficient ranged from 0.41 to 0.91 for low and from 1.10 to 2.10 for high MD, respectively. A Gaussian filter (3 mm half-width) was applied in accordance with observed TBI edema appearance. Only the MD maps were modified, the corresponding anatomical images remaining unmodified.

### TBI Patients

One patient (Supplementary Table 1 for inclusion and non-inclusion criteria) from each of 12 participating sites underwent an MRI exam (Supplementary Table 3 for details) between 5 and 13 days after trauma. At each site, additional DW images were acquired from 3 healthy volunteers (controls, see Supplementary Table 2 for inclusion and non-inclusion criteria) to compute reference site-dependent MD maps. The images from each site were anonymized, uploaded and stored in a dedicated centralized academic imaging data repository (shanoir.irisa.fr).

### Quality Control Procedure

**Figure 2:**
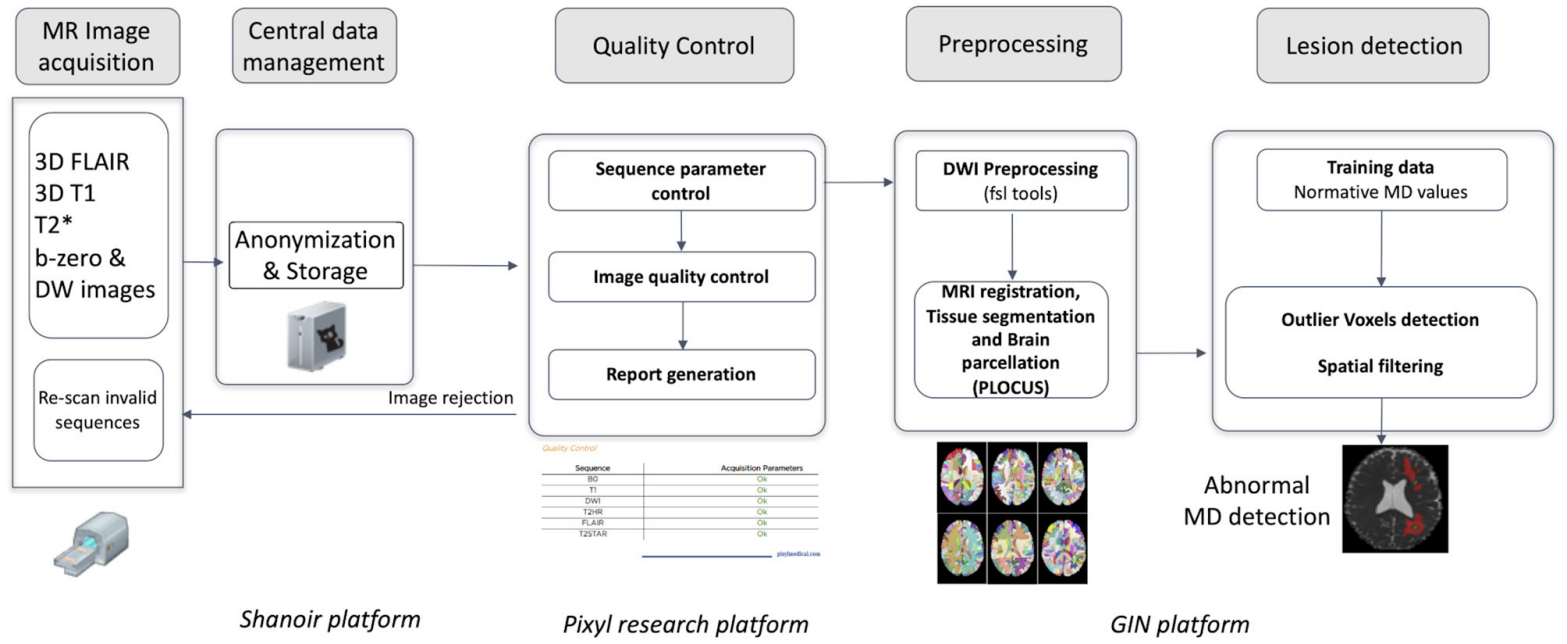
Image processing pipeline from image acquisition to automated detection of mean diffusivity abnormalities.

A quality control procedure was implemented to account for the high dependence of DWI on scanning equipment and acquisition protocol (13, 14). It was developed and deployed on the Pixyl (pixyl.ai) research platform (Figure 2). Automatic procedures analyzed specific Digital Imaging and Communications in Medicine (DICOM) tags, susceptibility artifacts, signal-to-noise ratio, motion artifacts, and corrupted slices. A quality control report was provided and validated by MR physicists (IT, CM).

### Manual Delineation

A panel of five expert neuroradiologists (AKa, AKr, DG, ES, and SK) manually annotated brain lesion areas from realistic TBI phantoms. The panel was unaware of the type, form and location of lesions that had been manually inserted. It was also blind to the nature of image, real or synthetic. Three of these experts (AKr, DG, and SK) then manually annotated brain lesion areas from images of TBI patients. They followed an annotation protocol based on DWI and the ITK-SNAP tool (http://www.itk.org) for annotation, blinded to each other and the ground truth. Other MRI sequences could be used for additional cues. To account for the inherent inter-rater variability in manual delineation (11), the Simultaneous Truth and Performance Level Estimation (STAPLE) method was used to provide an estimation of the rater consensus (10).

### Automated Quantification Procedure

Diffusion source images were denoised (15) and corrected for inter-volume subject motion and geometric distortion (Figure 2; Supplementary Material: Details of AQP); MD maps were computed from the trace of the diffusion tensor (see Supplementary Table 3). Brain was extracted and segmented using a Bayesian Markov Random Field approach named PLOCUS (16).

An automated atlas-based quantification procedure (AQP) was developed to partition the brain into defined regions, and to detect voxels with abnormal MD values, i.e., vasogenic and cellular edema, within those regions. AQP used six parcellation atlases to establish normative values and detect abnormal voxels according to the Potholes and Molehills method (12, 17). A voxel was considered as abnormal if its values deviated outside the normal range in ≥4 parcellation atlases. Voxels exhibiting high and low MD were considered if they formed part of a lesion of minimum size 0.16 and 0.12 ml, respectively. Voxels from within CSF or ventricles, as defined by segmentation of the T1-w sequence, were excluded. To deal with partial volume effects, abnormal high MD voxels at a distance of <3 mm from CSF voxels were also excluded. Lesion volume was expressed in ml and in brain volume fraction (%), the latter reflecting the ratio between brain lesion volume and supra-tentorial brain volume.

### Quantitative Comparison of the Manual and Automated Delineation Methods

Five spatial measurements were used to compare delineation methods: the Dice metric to measure the volume overlap, the Average Symmetrical Surface Distance (ASSD) to measure the average Euclidian surface distance, the Hausdorff Distance (HD) to measure the maximum distance between two surface points, and Precision and Recall (sensitivity) to assess over- and under-segmentation, respectively (see http://www.isles-challenge.org/ISLES2015/ for formulas). For ASSD and HD, expressed in mm, optimal values tend to 0. For Dice, Precision and Recall values, expressed within a 0–1 range, optimal values tend to 1.

### Statistical Analysis

Data were expressed as mean ± standard deviation or median (25–75th centiles). The Intra-class Correlation Coefficient (ICC) was used to compare the reliability of measurements between the rater consensus and AQP. The non-parametric Kruskall-Wallis test was used to compare spatial measurements obtained using GT, AQP, the rater consensus, and each rater (realistic phantom). The Mann-Whitney test was used to compare the rater consensus and AQP (TBI patients). Statistical significance was established when *P* < 0.05.

## Results

### Realistic TBI Phantoms

**Figure 3:**
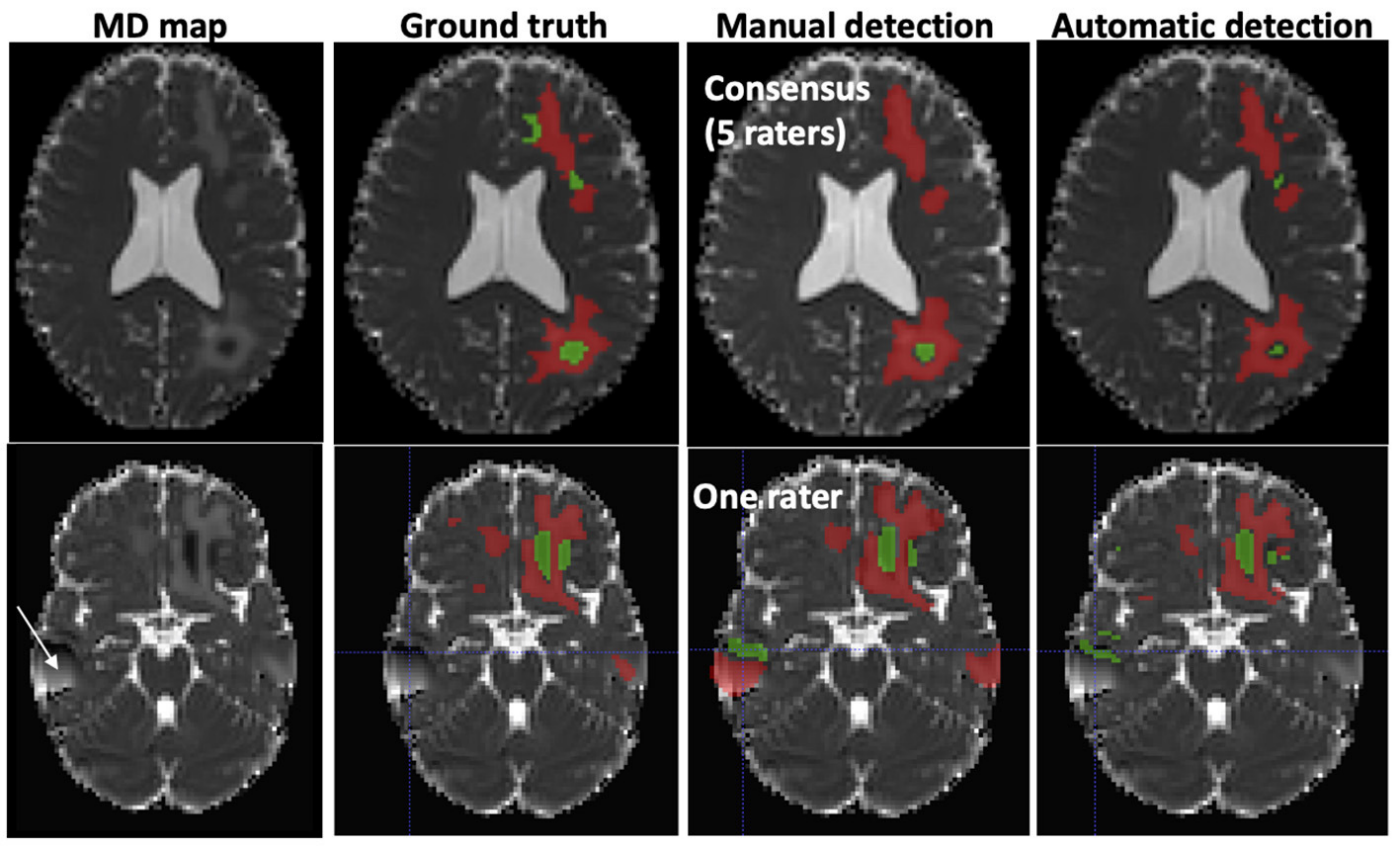
Typical examples of abnormal mean diffusivity (MD) values introduced in diffusion-weighted images (DWI) of two healthy volunteers (realistic TBI phantoms). **Top**: Good agreement between manual and automated segmentation. **Bottom**: Moderate agreement between manual and automated segmentation. The artifact (white arrow) was falsely detected as a lesion by one rater. Red, High MD values; Green, Low MD values.

The mean volume of the manually-inserted lesions was 31 ml, i.e., 2.2% of the total brain volume, and corresponded to the ground truth (GT). Typical examples of agreement between GT, manual delineation, and AQP are shown in Figure 3. AQP could detect additional lesions undetected by manual delineation and present in GT, and could exclude image artifacts. The time taken to process each case was 30 min for manual delineation vs. 10 min using AQP.

Dice and precision showed no significant difference between manual delineation and AQP (Dice: 0.75 and 0.72; and Precision: 0.66 and 0.70, respectively) (Table 1). The surface distance measurements of HD and ASSD were significantly higher using AQP compared to manual delineation (both *P* < 0.05).

**Table 1:** Spatial measurements for the 5 realistic TBI phantoms.

**Comparisons**	**Dice**	**HD (mm)**	**ASSD (mm)**	**Precision**	**Sensitivity**
Rater 1 vs. GT	0.75 (0.74 0.78)	6.7 (4.6 10.7)	0.5 (0.4 0.5)	0.69 (0.65 0.69)	0.88 (0.80 0.90)
Rater 2 vs. GT	0.74 (0.74 0.80)	8.5 (6.0 10.9)	0.6 (0.4 0.7)	0.74 (0.73 0.78)	0.74 (0.73 0.78)
Rater 3 vs. GT	0.71 (0.68 0.72)	12.4(10.9 18.5)	0.8 (0.6 0.8)	0.68 (0.66 0.70)	0.75 (0.68 0.76)
Rater 4 vs. GT	0.80 (0.76 0.81)	8.8 (7.1 17.8)	0.5 (0.3 0.7)	0.77 (0.74 0.84)	0.84 (0.79 0.85)
Rater 5 vs. GT	0.69 (0.66 0.72)	9.9 (9.2 10.6)	0.8 (0.7 0.8)	0.60 (0.55 0.64)	0.82 (0.78 0.85)
Rater consensus vs. GT	0.75 (0.74 0.80)	5.1 (4.1 10.7)	0.6 (0.4 0.6)	0.66 (0.65 0.72)	0.88 (0.87 0.90)
AQP vs. GT	0.72 (0.63 0.72)	24.6 (24.0 32.6)	1.4 (1.3 1.9)	0.70 (0.65 0.73)	0.75 (0.66 0.81)
AQP vs. rater consensus	0.63 (0.55 0.71)	25.3 (24.7 29.3)	1.8 (1.5 2.1)	0.74 (0.71 0.75)	0.59 (0.48 0.67)

*Each rater, rater consensus and automatic quantification procedure (AQP) were compared to the ground truth (GT) as reference. Data are expressed as median and 25–75th percentiles. Dice and precision obtained from rater consensus and AQP were comparable. HD and ASSD were higher using AQP compared to rater consensus (P < 0.05). HD, Hausdorff Distance; ASSD, Average Symmetrical Surface Distance*.

**Figure 4:**
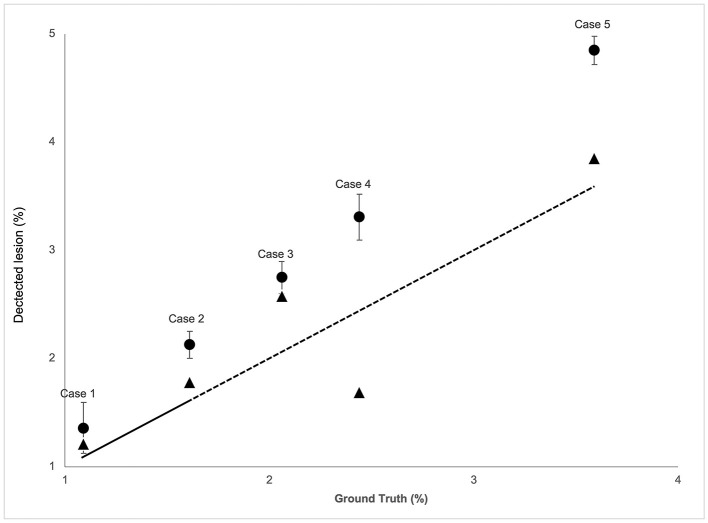
The correspondence analysis in brain lesion volume for the five realistic phantom cases for both the raters' consensus (circle) and AQP method (triangle) (*y*-axis) vs. the ground truth (*x*-axis). Total lesion volume (low + high MD) in % brain volume of diffusion-weighted images (mean, 95% confidence interval). The dashed line indicates the identity curve.

The lesion volumes corresponded to 2–4% of the brain volume, i.e., 18–40 ml. Both raters and AQP overestimated the lesion volumes in realistic phantoms, compared to GT (+32% for rater consensus; +13% for AQP) (Figure 4; Supplementary Table 4). Raters showed greater consistency with the ground truth, though tending to overestimate, as compared to AQP that demonstrated some jitter. The reliability between rater consensus ratings and AQP was moderate (ICC = 0.70) (Figure 4; Supplementary Table 4 and Bland-Altman plot in Supplementary Figure 1). Noteworthy is the overestimation of brain lesion volumes with high MD values by raters and underestimation of low MD lesion volumes. However, AQP, rater consensus and GT showed no significant differences regarding the determination of brain lesion volumes (*P* = 0.27).

### TBI Patients

**Figure 5:**
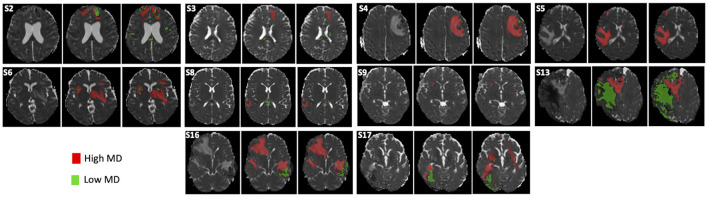
Delineation of brain lesions from diffusion-weighted images (DWI) in 10 TBI patients. The MD map **(left)**, rater consensus **(middle)** and automated quantification procedure **(right)** is shown for each patient. S2–S17 refer to the corresponding TBI subject (see Table 3).

**Figure 6:**
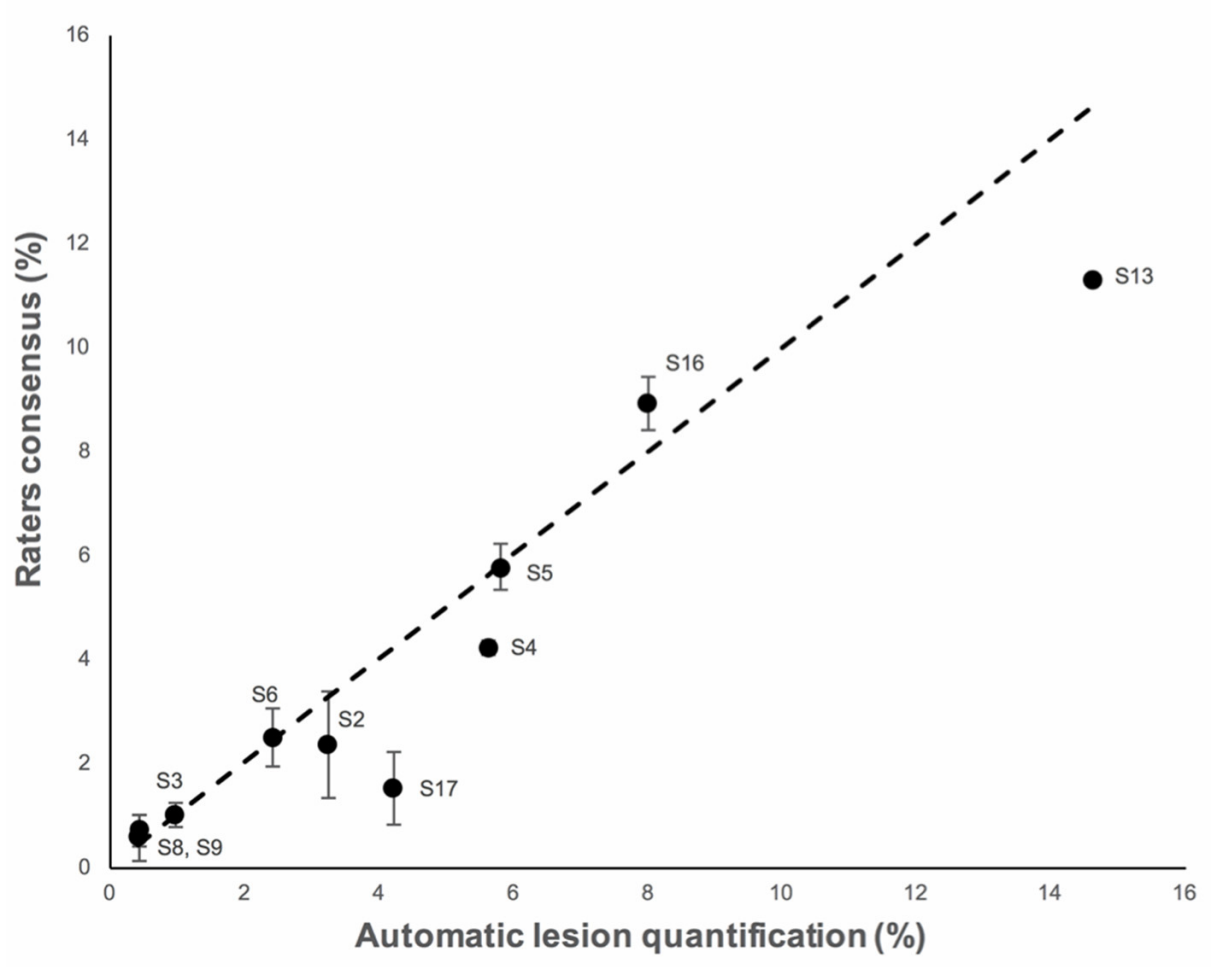
The correspondence analysis in brain lesion volume for the then TBI cases for both the raters' consensus (*y*-axis) and AQP method (*y*-axis). Total lesion volume (low + high MD) in % of the brain volume of diffusion-weighted images (mean, 95% confidence interval). The dashed line indicates the identity curve.

The characteristics of the patients are shown in Table 2. There was no significant difference in age between TBI patients and healthy volunters populations (*P* = 0.11). Two patients (#1 and #10) were excluded from the analysis because one rater delineated brain lesions visible on FLAIR images only. Figure 5 shows low and high MD brain lesions depicted by the rater consensus (middle) and by AQP (right). Additional brain lesions were found using AQP (cf. S2 and S17 in Figure 5). Dice, precision and sensitivity were comparable between AQP and rater consensus (Supplementary Table 5). HD and ASSD surface distance measurements were slightly different: median 28.8 and 2.0 mm for AQP vs. 19.6 and 1.4 mm for raters, respectively, with *P* < 0.02 for the former, non-significant for the latter (Supplementary Table 5). The brain lesion volumes of these patients computed by AQP ranged from 0.4 to 14.7% of the brain volume, i.e., 59 ml (19–84 ml), including 41 ml (14–72 ml) (median; 25–75th centiles) and 7 ml (5–17 ml) for high (vasogenic edema) and low (cellular edema) MD lesions, respectively. The reliability between manual and automated procedures was high (ICC = 0.92) (Figure 6; Supplementary Figure 2). The determination of brain lesion volumes by rater consensus and by AQP showed no significant differences (*P* = 0.91).

**Table 2:** Characteristics of the 12 patients with severe traumatic brain injury.

**Patients**	**Gender**	**Age (years)**	**Trauma to MRI delay (days)**	**Initial GCS**	**Type of MR scanner**
1	Male	31	5	5	Philips Achieva 3 T
2	Male	20	13	9	Siemens Skyra 3 T
3	Male	46	9	6	Siemens Avanto 1.5 T
4	Male	33	ND	ND	Siemens Skyra 3 T
5	Female	57	12	7	Siemens Aera 1.5 T
6	Male	30	13	6	Siemens Prisma 3 T
8	Male	21	5	3	Philips Achieva 3 T
9	Female	22	9	4	GE Signa 1.5 T
10	Male	50	13	5	GE 1Optima 0.5 T
13	Male	37	9	3	Siemens Skyra 3 T
16	Male	58	9	6	Siemens Aera 1.5 T
17	Male	71	13	6	Siemens Aera 1.5 T
Median (IQR)		35 (28; 52)	9 (8, 11)	6 (4, 6)	

*Patients #1 and #10 were excluded from the analysis because one rater delineated brain lesions on FLAIR images. GCS, Glasgow coma score; ND, not determined; IQR, interquartile range*.

**Table 3:** Spatial measurements for the 10 patients with severe traumatic brain injury.

**Patients**	**Dice**	**HD (mm)**	**ASSD (mm)**	**Precision**	**Sensitivity**	**AQP: Vol. edema (ml)**	**AQP: Vol. vasogenic edema (ml)**	**AQP: Vol. cellular edema (ml)**	**AQP: Vol. edema (%)**	**Raters consensus: Vol. edema (%)**
2	0.49	32.1	3.2	0.42	0.58	55.2	36.7	18.4	3.2	2.1
3	0.61	15.0	2.9	0.62	0.61	13.9	87.0	5.1.9	1.0	1.0
4	0.73	38.4	1.9	0.64	0.86	80.9	67.8	13.1	5.6	4.2
5	0.78	25.3	1.0	0.78	0.78	84.6	79.2	5.3	5.8	5.8
6	0.71	32.9	1.1	0.72	0.70	33.1	28.4	4.7	2.4	2.5
8	0.52	20.1	2.1	0.67	0.43	7.2	57.5	1.5	0.4	0.71
9	0.43	33.7	3.7	0.49	0.39	6.9	30.3	3.9	0.4	0.6
13	0.56	25.6	1.8	0.49	0.64	240.6	72.8	167.7	14.7	11.3
16	0.78	11.8	0.8	0.82	0.74	130.3	121.0	9.2	8.0	8.9
17	0.33	36.8	5.8	0.22	0.64	63.7	45.1	18.4	4.2	1.5
Median	0.58	28.8	2.0	0.63	0.64	59.4	41.0	7.3	3.7	2.4
(IQR)	(0.50; 0.72)	(21.4; 33.5)	(1.3; 3.1)	(0.49; 0.70)	(0.59; 0.73)	(19; 84)	(14; 72)	(5, 12)	(1.3; 5.7)	(1.3; 5.7)

*The automatic quantification procedure (AQP) is compared to the consensus from 3 raters. HD, Hausdorff Distance; ASSD, Average Symmetrical Surface Distance; IQR, interquartile range*.

For TBI patients, the ICC was higher between AQP and the rater consensus for high MD (0.97) than for low MD (0.48). Similarly, the inter-rater variability was smaller for high (6%) than for low (17%) MD.

## Discussion

Our fully automated procedure (AQP) provided findings in concordance with manually traced edematous brain lesions post-trauma. Based on both realistic digital phantoms and TBI patient MR images, AQP and the expert rater consensus provided comparable lesion volumes with abnormal MD values.

Even if the involvement of an expert is still necessary to control image quality and validate automated segmentation, the proposed approach is promising. Indeed, determining the type and volume of brain edema post-trauma using an accurate and automated approach could improve the management of severe TBI patients by directing precision-medicine-based treatment for optimal cerebral blood flow.

Limited MRI data exist on the type of brain injury in the acute phase after severe TBI (3, 4, 18). Pasco et al. explored the type of post-traumatic brain edema in the white matter using manual delineation of ROIs based on apparent diffusion coefficient (ADC) values (3). In the present study, we confirmed that both types of brain edema could be found at the early phase of severe TBI. Their quantitative automated distinction could be of interest in terms of clinical management: while a predominance of lesions with cellular edema (low MD), reflecting brain ischemia, would favor the maintenance of high levels of cerebral perfusion pressure (CPP; with CPP = mean arterial blood pressure – intracranial pressure); lower levels of CPP would be preferable with vasogenic edema lesions (high MD) where a disruption of the blood-brain barrier is predominant.

Few studies have explored a fully automated approach to delineate TBI brain lesions. Segmentation methods such as Siena, applied to T1-weighted images, misclassified focal TBI lesion in gray matter (19). Using a deep learning approach, Kamnitsas et al. found 0.63 and 0.68 for Dice and precision, respectively (20). Better results were obtained (21) using a modified version of the Inception architecture (22). Our approach permitted the quantification of cellular and vasogenic volumes, as reflected by low and high MD values, and required no training phase with a large set of manual annotations such as is required for deep learning approaches. The training phase in our approach is solely based on establishing normal MD distributions for healthy volunteers in each center.

Automated AQP and rater delineation showed interesting differences. For one, as seen in Figures 3, 5, additional brain lesions were found using AQP. Moreover, the contours of the manually-traced ROI were smoother and less detailed than those of the AQP. While these differences had negligible impact on the estimated brain lesion volumes and on the spatial overlap measures (Dice), they can explain the differences in HD, a measure of the maximum distance between two surface points.

Regarding lesion volumes, each manually traced lesion volume was overestimated (33% on average for phantoms) compared to AQP (5% on average). A closer look at the data shows that the manual delineation systematically overestimated the volume of high MD lesion (44 vs. 7% for AQP on average for phantoms). We observed also that Dice similarity coefficient and precision for automated and manual methods were low (between 0.59 and 0.70) compared to values obtained for stroke or brain tumor. These low values are indicative of the difficulty in manual delineation of trauma lesions, even for experts, and may also explain the high level of variability among the manual delineation values (16% for phantoms and 12% for patients).

The aim of our study was to determine whether automatic quantification of brain lesions would be as accurate as manual delineation in two situations: phantom images and TBI images. The latter obviously reflect the real life with possible presence of blood and tissue deformation. In spite of that, the results obtained with the proposed approach are encouraging. However, it is important to note that the study of TBI patient management, and associated imaging support, is inherently challenging. As such, the authors draw attention to several limitations. First, brain lesions of realistic TBI phantoms were inserted in brain MD maps only. The use of TBI phantoms with multiparametric images might have resulted in a better agreement with GT. Second, normative MD values were obtained using a limited sample of only 3 young male volunteers per site and TBI data from one patient per site. Although it is important to consider sources of variability between patients and volunteers, the reliability between manual and automated procedures was nevertheless high for TBI patients. Third, we considered one type of MR sequence (diffusion) and one metric (MD) for detecting the presence of vasogenic and cellular edema. Indeed, MD has been chosen because it is widely used to determine the volume of ischemic tissue (8). Brain ischemia is one leading cause of secondary brain damage after severe TBI (23) and can result in cellular edema and/or vasogenic edema in case of brain blood barrier disruption. We did not consider hemorrhagic brain lesions such as contusions, subdural and extradural hematomas, subarachnoid hemorrhage and petechiae, although some may have appeared as low MD lesions. Fourth, while the approach seems robust to artifacts (see Figure 3), whether it misinterprets some as lesions warrants further investigation. Fifth, a larger panel of experts could offer more statistical weight to the results, although it should be noted that we employed the largest panel (5) so far of experts in TBI imaging, according to the literature (5, 6). Sixth, a more comprehensive patient dataset to correlate the volume of brain lesions in TBI patients with their outcome was not available.

In conclusion, an automated atlas-based quantification procedure has been effectively shown to quantify the volume of low and high MD brain lesions after trauma, and thus allow the determination of the type and volume of edematous brain lesions. This approach had comparable performance with manual delineation by a panel of experts. It will be used in a large cohort of patients enrolled in the multicenter OxyTC trial (NCT02754063). We will see whether the quantification of brain lesion volume as well as type and location may play a role in the neurologic outcome after severe TBI.

## Data Availability Statement

The MR data supporting the results of this study are available from the corresponding author upon reasonable request.

## Ethics Statement

The studies involving human participants were reviewed and approved by the local ethical committee (OxyTC, NCT02754063). All participants or the next of kin provided written informed consent to participate to the study.

## Author Contributions

AKa, AKr, DG, ES, and SK: manual lesion delineation. CM, FF, PR, SD, and AT: conceptualization, segmentation method development, and data analysis. IT: data acquisition and recruitment supervision. TM: manual lesion delineation, data analysis, and writing-review and editing. EB: conceptualization, methodology, data analysis, resources, and writing-review and editing. MD: conceptualization, methodology, data analysis, resources, writing-original draft, and writing-review and editing. J-FP: overall project supervision, resources, funding acquisition, and writing-review. All authors contributed to the article and approved the submitted version.

## Funding

CM was the recipient of a grant from the Gueules Cassées foundation. The authors would like to thank the French network REMI for its assistance in the homogenization of the MR acquisition protocols across all imaging centers (France Life Imaging, grants C7H-FLI11B23 and C7H-FLI11B19). Grenoble MRI facility IRMaGe was partly funded by the French program Investissement d'avenir run by the Agence Nationale de la Recherche: grant Infrastructure d'avenir en Biologie Santé ANR-11-INBS-0006.

## Conflict of Interest

The authors declare that the research was conducted in the absence of any commercial or financial relationships that could be construed as a potential conflict of interest.

## Publisher's Note

All claims expressed in this article are solely those of the authors and do not necessarily represent those of their affiliated organizations, or those of the publisher, the editors and the reviewers. Any product that may be evaluated in this article, or claim that may be made by its manufacturer, is not guaranteed or endorsed by the publisher.

## Abbreviations

Average Symmetrical Surface Distance (ASSD)
AQP: Automated Quantification Procedure
DWI: Diffusion Weighted Imaging
GT: Ground Truth
Hausdorff Distance (HD)
Intra-class Correlation Coefficient (ICC)
MD: Mean Diffusivity
SM: Supplementary Material
TBI: Traumatic Brain injury
