# Supplementary material for: Automated Quantification of Brain Lesion Volume From Post-trauma MR Diffusion-Weighted Images
Source: Front Neurol. 2022 Feb 23;12:740603. doi: 10.3389/fneur.2021.740603 (PMC8905597; doi:10.3389/fneur.2021.740603)
Supplement: Supplementary file 1 [file Data_Sheet_1.PDF]

Supplementary Material

Supplementary methods – Details of AQP

During the automated quantification procedure (AQP) (**Fig. 2**), diffusion source images were denoised (Manjon et al., 2013) and hypo or hyper-intense slices were automatically detected. Corrupted slices were corrected by interpolating adjacent slices. Diffusion source images were corrected for inter-volume subject motion and geometric distortion due to susceptibility and eddy current using FMRIB Software Library (FSL) (http://www.fmrib.ox.ac.uk/fsl/) and combined to form diffusion-weighted images. Then, MD maps were computed from the trace of the diffusion-weighted tensors (30 directions, see Table 3). Brain extraction was performed by the ROBEX software (https://sites.google.com/site/jeiglesias/ROBEX) using FLAIR, T2* and T1-weighted images. After registration to the corresponding diffusion-weighted image, brain tissue and CSF were segmented using a Bayesian Markovian approach named PLOCUS (Doyle et al., 2012). The Montreal Neurological Institute atlas, i.e. MNI152 standard-space T1-weighted average structural template image, was used as *a priori* probabilistic knowledge of tissue localization. Combined with the brain mask provided by ROBEX, this atlas permitted accounting for the post-TBI skull deformation. The total brain volume corresponded to the supra-tentorial volume computation.

The AQP used a multi-atlas approach to detect voxels the values of which deviated from normative values, according to the Pothole and Molehills method (Watts et al., 2014). This multi-atlas approach was based on four atlases: Neuromorphometrics atlas (http://www.neuromorphometrics.com/), HarvardOxford atlas (FSL), Desikan atlas (FreeSurfer), and ICBM DTI81 atlas, which together allowed the production of 6 brain parcellations corresponding to 1402 regions of interest (ROIs). Using these parcellations and the MRI data of volunteers, a normative MD value was computed for each ROI. For TBI realistic phantoms and TBI patients, the MD value of each voxel was compared to the 6 normative MD values. Abnormal MD values were considered where MD was below the 2nd percentile of normative MD values or above the 94.8^{th} percentile. The asymmetry in thresholds is due to the

skewness of the MD distribution. Calculations were made for each of the 6 parcellations. A voxel was finally considered as abnormal if 4/6 parcellations at least were found outside the normal range. Thereafter, brain lesion was considered where 20 (or 15) contiguous abnormal voxels at least were observed with high (or low) MD values, respectively, corresponding to a minimal volume of 0.160 (0.120) ml, respectively. Voxels labeled as CSF or ventricles were excluded. To deal with the partial volume effect, abnormal voxels at a distance of less than 3 mm from CSF voxels were also excluded. Lesion volume was expressed either in ml or as a brain volume fraction (%), the latter of which reflects the ratio between brain lesion volume and supra-tentorial brain volume.

References

Doyle, S., Forbes, F., and Dojat, M. (Year). "P-LOCUS, a complete suite for brain scan segmentation", in: *9h IEEE International Symposium on Biomedical Imaging (ISBI)*).

Manjon, J.V., Coupe, P., Concha, L., Buades, A., Collins, D.L., and Robles, M. (2013). Diffusion weighted image denoising using overcomplete local PCA. *PLoS One* 8(9), e73021.

Watts, R., Thomas, A., Filippi, C.G., Nickerson, J.P., and Freeman, K. (2014). Potholes and molehills: bias in the diagnostic performance of diffusion-tensor imaging in concussion. *Radiology* 272(1), 217-223.

SM Table 1: Inclusion and non-inclusion criteria for patients

Inclusion Criteria

- Age between 18 and 75 years
- Severe non-penetrating TBI (initial GCS 3-8) with motor GCS between 1 and 4
- Possible associated extracranial lesions, except tetraplegia
- Monitoring within the first 16 hours after primary traumatic injury
- Indication for ICP monitoring on admission as part of the management
- Indication for continuous sedation/analgesia for more than 48 hours
- Under mechanical ventilation with stable conditions
- Affiliation to the French social security health care insurance or similar system of an EU member state, Norway, Lichtenstein, Iceland or Switzerland.
- French-speaking

No inclusion criteria

- Penetrating TBI
- GCS 3 with bilateral fixed dilated pupils
- Decompressive craniectomy prior to enrolment
- Contraindication of ICP and/or PbtO2 monitoring
- Persistent hemodynamic or respiratory instability
- Hypothermia <34°C on admission
- Venous or arterial lactate concentration >5 mmol/l at randomization
- Life expectancy < 24 hours
- Cardiac arrest at initial presentation
- Tetraplegia
- Neuropsychiatric co-morbidities that could interfere with 6- and 12-month evaluations
- Consent refusal
- Pregnancy
- Participation in another therapeutic study with written consent
- Inability to attend the 6-month follow-up
- Permanent contraindications to MRI
- Ischemic stroke after carotid artery dissection
- Incapacitated patients in accordance with article L 1121-5 to L1121-8 of the public health code.

SM Table 2: Inclusion and non-inclusion criteria for Controls

Inclusion criteria:

- Male between 18 and 60
- No history of chronic disease or brain trauma
- Written informed consent
- Affiliated to the French social security system

Non-inclusion criteria:

- Contra-indications to MRI
- Claustrophobia

SM Table 3: Main MR acquisition parameters for the different scanners IT. NSA: Number of acquisitions. Na: Not available.

3T	3D FLAIR		3D T1 MPRAGE		T2*		DWI		b0	
	Philips	Siemens	Philips	Siemens	Philips	Siemens	Philips	Siemens	Philips	Siemens
Orientation	sagittal		sagittal		transversal		transversal		transversal	
TR / TE (ms)	4800/390	5000/402	2500/3.3	2300/2	2200/16	1110/12	9800/80	10900/80	9800/80	10900/80
TI (ms)	1650	1800	940	900						
Turbo / EPI	182	270	232	208			Single shot EPI		Single shot EPI	
Resolution (mm^3)	1x1x1		1x1x1		1x1x2		2x2x2		2x2x2	
Flip angle	90°	120° variable	9°		16°	20°	90°		90°	
Slices	180	176	180	176	70	66	70		70	
Gap (mm)	0		0		0		0		0	
NSA	2	1	1		1		1		1	
Diffusion direction							1 b=0<br>32 b=1000	1 b=0<br>30 b=1000	1 b=0	
Phase direction							P >> A		A >> P	

1.5T	3D FLAIR		3D T1 MPRAGE		T2*		DWI		b0	
	GE	Siemens	GE	Siemens	GE	Siemens	GE	Siemens	GE	Siemens
Orientation	sagittal		sagittal		transversal		transversal		transversal	
TR / TE (ms)	8000/180	5000/335	6.2/2	2000/2.9	720/23	1230/20	12000/85	6700/85	12000/85	6700/85
TI (ms)	2100	1800	450	1100						
Turbo / EPI	213	242	?	208			Single shot EPI		Single shot EPI	
Resolution (mm^3)	1x1x1		1x1x1		1x1x3		2.5x2.5x2.5		2.5x2.5x2.5	
Flip angle	Na	120°	12°	15°	20°		90°		90°	
Slices	156	176	160	160-192	48	44	55		55	
Gap (mm)	Na	0	0		0		0		0	
NSA	2	1	1		2	1	1		1	
Diffusion directions							1 b=0<br>30 b=1000		1 b=0<br>30 b=1000	
Phase direction							P >> A		A >> P	

SM Table 4: Volume, expressed as a % of the brain volume, of the lesion for each realistic TBI phantom as estimated using the automated method, by each rater and by the STAPLE consensus of the five raters' scores.

Difference in lesion volume is expressed as a % of the GT (Ground Truth) lesion. AQP: automated quantification procedure.

Case	GT Vol (%)	AQP	Rater 1	Rater 2	Rater 3	Rater 4	Rater 5	Rater consensus	AQP-GT (%)	Rater consensus-GT (%)
1	1.1	1.2	1.4	1.0	1.1	1.03	1.7	1.4	11.0	24.8
2	1.6	1.8	2.2	1.5	1.9	1.8	1.9	2.1	10.6	32.3
3	2.1	2.6	2.5	2.0	2.5	2.2	2.9	2.7	25.2	33.5
4	2.4	1.7	3.2	2.5	2.6	2.0	3.5	3.3	-30.7	35.7
5	3.6	3.8	4.1	3.6	3.8	4.4	5.0	4.8	7.2	35.1

SM Table 5: All mean spatial measurements for the 10 TBI patients estimated by each rater. For each rater, the ground truth (GT) was computed using the STAPLE method based on the results of the two other raters.

Median [25th, 75th].

Comparison	Dice	Hausdorff distance (mm)	ASSD (mm)	Precision	Sensitivity
Rater 1 vs GT	0.56 [0.46 0.74]	20.4 [15.6 27.6]	1.5 [0.9 2.2]	0.42 [0.32 0.69]	0.82 [0.75 0.89]
Rater 2 vs GT	0.58 [0.40 0.67]	21.6 [16.5 26.5]	1.3 [1.0 2.1]	0.44 [0.27 0.59]	0.84 [0.80 0.89]
Rater 3 vs GT	0.67 [0.54 0.74]	16.6 [9.1 19.6]	1.14 [0.7 1.8]	0.67 [0.65 0.75]	0.70 [0.58 0.76]
Median	0.59 [0.44 0.74]	19.6 [13.6 26.1]	1.4 [0.8 2.2]	0.60 [0.33 0.71]	0.79 [0.70 0.88]

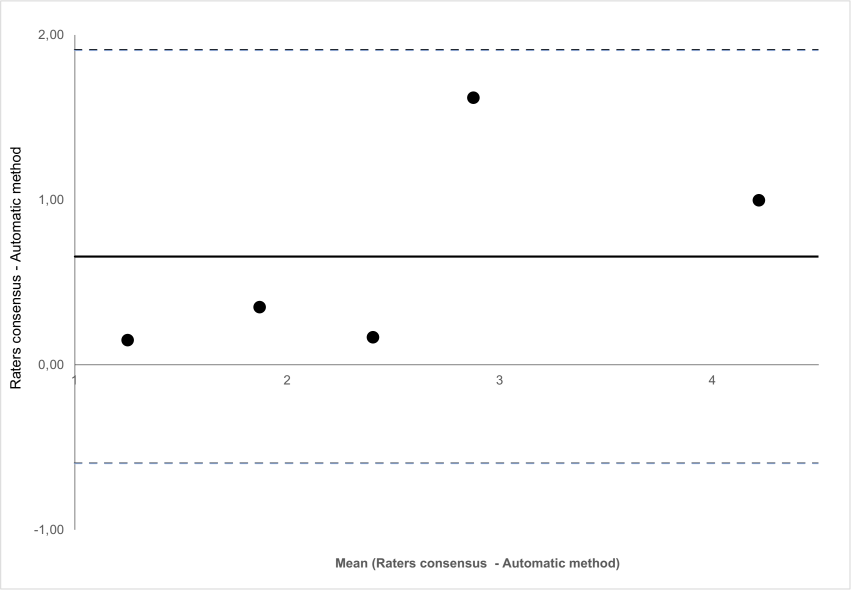

Fig. 1: Bland Altman plot for the five realistic phantom cases. Y-axis: Difference between the raters' consensus and AQP method. X-axis: Mean between the manual consensus and automatic method. The dashed lines indicate +/- 1.96 SD.

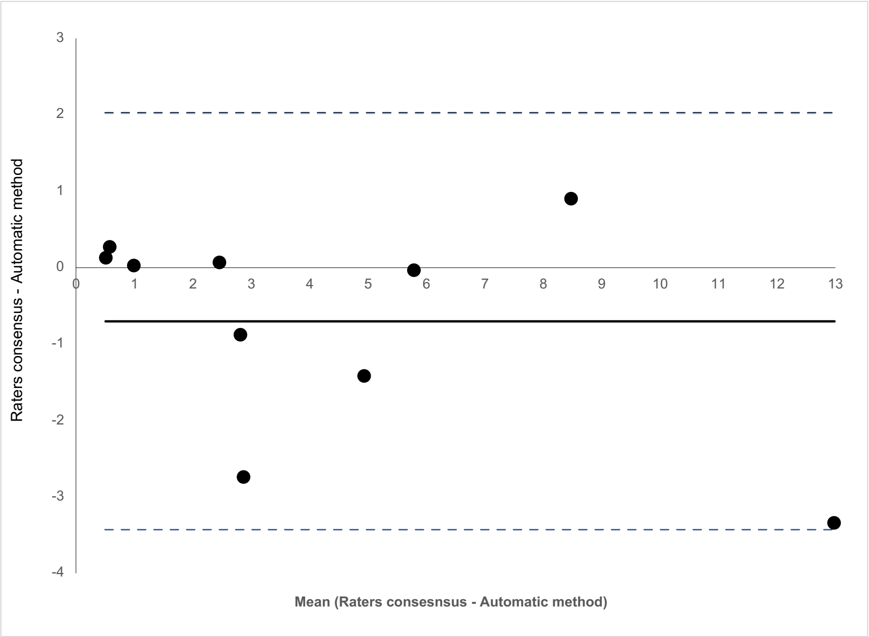

Fig. 2: Bland Altman plot for the ten TBI patient cases. Y-axis: Difference between the raters' consensus and AQP method. X-axis: Mean between the manual and automatic method. The dashed lines indicate +/- 1.96 SD.
